# Hydrothermal Magnesium Alloy Extracts Modulate MicroRNA Expression in RAW264.7 Cells: Implications for Bone Remodeling

**DOI:** 10.3390/jfb16080303

**Published:** 2025-08-21

**Authors:** Viviana Costa, Lavinia Raimondi, Daniele Bellavia, Angela De Luca, Pasquale Guglielmi, Angela Cusanno, Luca Cattini, Lia Pulsatelli, Matteo Pavarini, Roberto Chiesa, Gianluca Giavaresi

**Affiliations:** 1 Surgical Sciences and Technologies-SS Omics Science Platform for Personalized Orthopedics, IRCCS Istituto Ortopedico Rizzoli, 40136 Bologna, Italy; viviana.costa@ior.it (V.C.); daniele.bellavia@ior.it (D.B.); angela.deluca@ior.it (A.D.L.); gianluca.giavaresi@ior.it (G.G.); 2 Department of Mechanics, Mathematics and Management, Politecnico University of Bari, 70125 Bari, Italy; pasquale.guglielmi@poliba.it (P.G.); angela.cusanno@poliba.it (A.C.); 3 Laboratory of Immunorheumatology and Tissue Regeneration, IRCCS Istituto Ortopedico Rizzoli, Via di Barbiano 1/10, 40136 Bologna, Italy; luca.cattini@ior.it (L.C.); lia.pulsatelli@ior.it (L.P.); 4 Department of Chemistry, Materials and Chemical Engineering ‘G. Natta’, Politecnico di Milano, 20135 Milan, Italy; matteo.pavarini@polimi.it (M.P.); roberto.chiesa@polimi.it (R.C.)

**Keywords:** Mg alloy, bone healing, osteoclast differentiation, miRNAs

## Abstract

Magnesium (Mg) alloys, particularly Mg AZ31, have emerged as promising biomaterials for orthopedic applications due to their biodegradability and favorable mechanical characteristics. Among these, the Mg AZ31+SPF alloy, subjected to hydrothermal (HT) treatment, has demonstrated enhanced bioactivity. Our previous research established that this surface modification supports the osteogenic differentiation of human mesenchymal stem cells (hMSCs) by modulating both canonical and non-canonical signaling pathways, including those implicated in osteogenesis, hypoxic response, exosome biogenesis, and lipid metabolism. In the present study, we extended our investigation to assess the effects of Mg AZ31+SPF+HT and Mg AZ31+SPF extracts on murine pre-osteoclasts (RAW 264.7 cells) over 3- and 6-day treatment periods. The primary objectives were to evaluate biocompatibility and to investigate potential impacts on osteoclastogenesis induction and miRNA expression profiles. Methods: To assess cytocompatibility, metabolic activity, DNA integrity, and morphological alterations in RAW 264.7 cells were evaluated. Osteoclast differentiation was quantified using TRAP staining, alongside the assessment of osteoclastogenic marker expression by qRT-PCR and ELISA. The immunomodulatory properties of the extracts were examined using multiplex BioPlex assays to quantify soluble factors involved in bone healing. Additionally, global miRNA expression profiling was performed using a specialized panel targeting 82 microRNAs implicated in bone remodeling and inflammatory signaling. Results: Mg AZ31+SPF+HT extract exhibited high biocompatibility, with no observable adverse effects on cell viability. Notably, a significant reduction in the number of TRAP-positive and multinucleated cells was observed relative to the Mg AZ31+SPF group. This effect was corroborated by the downregulation of osteoclast-specific gene expression and decreased MMP9 protein levels. Cytokine profiling indicated that Mg AZ31+SPF+HT extract promoted an earlier release of key cytokines involved in maintaining the balance between bone formation and resorption, suggesting a beneficial role in bone healing. Furthermore, miRNA profiling revealed a distinct regulatory signature in Mg AZ31+SPF+HT-treated cells, with differentially expressed miRNAs associated with inflammation, osteoclast differentiation, apoptosis, bone resorption, hypoxic response, and metabolic processes compared to Mg AZ31+SPF-treated cells. Conclusions: Collectively, these findings indicate that hydrothermal treatment of Mg AZ31+SPF (resulting in Mg AZ31+SPF+HT) attenuates pre-osteoclast activation by influencing cellular morphology, gene and protein expression, as well as post-transcriptional regulation via modulation of miRNAs. The preliminary identification of miRNAs and the activation of their regulatory networks in pre-osteoclasts exposed to hydrothermally treated Mg alloy are described herein. In the context of orthopedic surgery—where balanced bone remodeling is imperative—our results emphasize the dual significance of promoting bone formation while modulating bone resorption to achieve optimal implant integration and ensure long-term bone health.

## 1. Introduction

Magnesium-based alloys are increasingly explored in orthopedic biomaterials due to their biodegradability and mechanical compatibility with bone. Despite their prevalence in the human body, Mg alloys utilized in prosthetic implants exhibit a high degree of degradation and the release of H+ ions, which modify the pH of biological fluids, thereby inducing a chronic inflammatory process [1] in the site of implant and, consequently, its implant failure.

To mitigate these deleterious effects, a range of coating methods have been developed with the precise aim of enhancing the biocompatibility and osteointegrative capacity of these alloys. One such method is hydrothermal treatment (HT), which has been previously described by us as improving viability and inducing osteogenic differentiation in an in vitro model of hMSCs [2,3].

However, it is argued that investigating its effects only on an in vitro model evaluating hMSC-induced osteogenesis is a limitation. In fact, in the site of fracture, Mg alloys normally interact with hMSCs, immune cells, endothelial cells, osteogenesis-related cells, nerve cells, etc., as well as with osteoclasts and bone marrow monocyte–macrophage (BMM) precursors, which are responsible for the initial step of bone resorption [4,5]. The bone remodeling process induced by magnesium alloys should trigger signaling from pre-osteoblasts and pre-osteoclasts, allowing regeneration of the surface. Under physiological conditions, the osteoclastogenesis process is also regulated by osteoblasts and stromal cells that enrich the microenvironment with the secretion of macrophage colony-stimulating factor (M-CSF)—which plays a crucial part in the survival, growth, and expression of RANK in early OCPs (monocyte/macrophage lineage) [6,7]—and the receptor activator of nuclear factor NF-KB ligand (RANKL), which mediates the promotion of multiple signal transduction pathways (NF-Kβ, etc.). It has been demonstrated that during the processes of repairing/healing fractures, Mg alloys regulate the response and interactions of multiple cells involved in bone healing [8,9]. The impact of magnesium alloy implants on the activation of pre-osteoclasts is a relatively new area of research. Only a few studies have been conducted so far [10,11]. In order to enhance understanding of the mechanism activated by Mg alloy implants in vivo, this in vitro study opens a new perspective on their possible involvement in the modulation of different microRNAs. MicroRNAs (miRNAs) are a class of small, noncoding RNAs of 19–25 nucleotides that, after binding to 3′-untranslated regions (3′-UTR) within a target mRNA, induce its degradation or silencing. An increasing number of miRNAs have been identified as participating in osteoclast formation, differentiation, apoptosis, and resorption [12,13,14] under physiological and pathological conditions, but no information regarding the effects of Mg alloys on pre-osteoclast activation has been reported [15], especially not about the miRNAs induced by the hydrothermal treatments of Mg AZ31+SPF. The evidence obtained by a clinical study conducted on patients involved in orthopedic surgery, such as for joint replacement or fracture repair, underlined the importance of circulating miRNAs, released by exosomes or vesicles, in biological fluids as biomarkers of the state of “bone healing” [16,17,18]. Regarding this, in our recent study, it was demonstrated that the hydrothermal treatment of Mg AZ31+SPF is able to induce an increase in the secretion of proteins involved in exosome formation or post-transcriptional modification in a hMSC model [19,20], suggesting the role of hydrothermal treatment as an activator of specific cell–cell communication at the site of implant.

In light of this observation, the present study was designed to investigate the impact of hydrothermal processing of Mg AZ31+SPF on pre-osteoclast cells within the niche of the implant, and to preliminarily identify a possible biomarker of prosthetic implant integration. To achieve this aim, herein, we investigated the effects of Mg alloy extracts on an in vitro model of pre-osteoclast RAW 264.7 cells, analyzing the viability response and osteoclast–gene and protein activation, and identifying a set of differentially expressed miRNAs between Mg AZ31+SPF- and Mg AZ31+SPF+HT-treated cells, prompting us to hypothesize about their potential clinical implications. Furthermore, the potential involvement of specific microRNAs in the success of an effective prosthetic implant remains to be fully elucidated.

## 2. Materials and Methods

### 2.1. In Vitro Models of Murine Pre-Osteoclast Cells

RAW 264.7 cells were purchased from ATCC^®^ and cultured at 37 °C and 5% CO_2_ in DMEM supplemented with 10% FBS, 2 mM glutamine, and antibiotics (100 U/mL penicillin, 100 μg/mL streptomycin). For the in vitro treatments, RAW 264.7 were seeded onto 12-well plates at a concentration of 25,000/well in the presence of Mg alloy extracts obtained as described by Tatullo et al. [2] using the dilution and method identified by De Luca et al. (25% extracts in 75% DMEM) [3]. Briefly, the Mg disks were immersed in an extraction vehicle composed of 50% PBS (w/o Ca^2+^ and Mg^2+^) and 50% DMEM. Samples were incubated at 37 °C in a 5% CO_2_ atmosphere for 72 h under static conditions. After recovery of the supernatant and centrifugation to remove the matter, the samples were collected. To determine the optimal extraction vehicle percentage, the in vitro test was performed. The viability results demonstrated that the 25% dilution of the extraction vehicle ensured a minimum viability of at least 80%, which is the safety level according to ISO 10993-5 [3]. RAW 264.7 cells were induced to differentiate into osteoclast cells through treatment with 25 ng/mL of human recombinant RANK Ligand (Gibco, Life Technologies, Frederick, MD, USA) for 3 and 6 days to perform viability assays and protein release evaluations, and for 6 days for gene expression analysis. The medium was changed every three days.

For the in vitro test, we divided the sample into 4 groups: Ctrl- for untreated RAW cells; Ctrl+ for RAW cells treated with hrRANKL; Mg AZ31+SPF for RAW cells treated with Mg AZ31+SPF extracts; Mg AZ31+SPF+HT for RAW cells treated with Mg AZ31+SPF+HT extract. After 3 and 6 days of culture, the samples were recovered and in vitro-tested.

### 2.2. RAW 264.7 Cell Viability (WST-1 Test)

To assess cell viability, the WST-1 colorimetric reagent (Roche Diagnostics GmbH, Mannheim, Germany) was utilized. The assay was performed by adding WST-1 reagent (10% *vol*/*vol*) to the cell monolayer in each well (seeded as reported below). The formazan dye produced by viable cells was quantified spectrophotometrically at the absorbance of 450 nm using the Bio-Rad Microplate Reader (Bio-Rad Laboratories, Hercules, CA, USA) after 4 h of incubation. The results are reported as the mean value of absorbance at 450 nm (n = 3 duplicates).

### 2.3. dsDNA Concentration (PicoGreen Assay)

Quantification of the dsDNA content was performed using a fluorimetric Quant-iT PicoGreen dsDNA Assay Kit (Invitrogen™, Life Technologies-EuroClone S.p.A, Pero-Milan, Italy). Following a thorough washing step with phosphate-buffered saline, 250 μL of lysis solution was added to each well plate, and cell lysis was then induced through three cycles of freezing–thawing. Following a five-minute incubation at room temperature and protected from light, the dsDNA content was calculated from the lysates by adding 100 μL of fluorescent nucleic acid stain to each scaffold. Fluorescence was measured using a GloMax multiwell plate reader (GloMax, Promega Corporation, Madison, WI, USA). Data are reported as the values of fluorescence in each group (n = 3 duplicates).

### 2.4. RNA Extraction and Real-Time PCR

Total RNA was extracted from the RAW 264.7 after 6 days of treatment using a commercially available illustraRNAspin Mini Isolation Kit (GE Healthcare, Milan, Italy), according to the manufacturer’s instructions. RNA was reverse transcribed to cDNA using a HighCapacity cDNA Reverse Transcription Kit (Applied Biosystems, Fisher Scientific Italia, Segrate, Italy), and relative real-time PCR was performed in duplicates for each data point using the QuantiTect Primers (Quiagen Srl, Venlo, The Netherlands) QT00131012 Mm_Acp5_1_SG, QT00150703 Mm_Ctsk_1_SG, and QT01136772 Mm_Actb_2_SG. Changes in the target mRNA content relative to the housekeeping β-actin gene were determined using the 2^−ΔΔCt^ method [21].

Isolation of miRNAs was performed using a commercial kit (RNeasy Plus MicroKit Qiagen), and reverse transcription was performed using a miRCURY LNA RT Kit (Qiagen). MiRNA expression was evaluated using miRNA Focus PCR Panel Mouse Apoptosis Focus V2 (Qiagen) following the instructions reported in the datasheet. The analysis of miRNA expression between the experimental groups was conducted using the Gene Globe analysis web through the representation of fold change 2^−ΔCt^ and bioinformatic evaluation using miRNet and Enrichr tools. For the definition of the significant expression of miRNAs identified, a 2-fold modulation between the tested groups and a *p* < 0.05 limit was used (CT cut-off: 35; *p*-value threshold: 0.05; fold-regulation threshold: 2).

### 2.5. Evaluation of Supernatant Soluble Factors

The concentrations of soluble factors were evaluated using a multiplex bead immunoassay. Samples were assayed for chitinase 3-like 1, procollagen type I α1, LRG1, serpin, fibronectin, tumor necrosis factor alpha (TNFα), interferon gamma (IFNγ), and transforming growth factor (TGF)-β1 and -β2 concentrations. These were simultaneously evaluated using commercially available bead-based sandwich immunoassay kits (Luminex Performance Assay Multiplex kits, R&D Systems, Minneapolis, MN, USA), following the manufacturer’s instructions. The concentrations of the samples were then determined by interpolation from the standard curves. The data are reported as fold of induction (FOI) compared to the untreated cells (Ctrl-).

### 2.6. TRAP Staining Assay

RAW 264.7 cells were seeded at a density of 5 × 10^4^ cells in 12-well plates and cultured in OC medium (DMEM hg, 10% FBS, and hrRANKL (25 µg/mL)) or with Mg AZ31+SPF or Mg AZ31+SPF+HT extract (diluted into DMEM hg and 10% FBS) for 6 days. Cells were stained for detection of tartrate-resistant acid phosphatase (TRAP) activity, according to the manufacturer’s protocol (Acid Phosphatase, Leukocyte (TRAP) Kit; Sigma-Aldrich, St. Louis, MO, USA) and evaluated via light microscopy. Multinucleated TRAP+ cells containing more than three nuclei were scored as mature osteoclasts. Two independent experiments were performed in triplicate; cells from 5 different fields were counted for each condition.

### 2.7. ELISA Assay

RAW 264.7 cells were treated as described above, and secreted MMP9 (matrix metalloproteinase 9) was quantified using a mouse MMP9 ELISA assay (Wuhan Fine Biological Technology Co., Ltd., Wuhan, China), according to the manufacturer’s instructions. Data are expressed as fold of induction (FOI) compared to the Ctrl- group (n = 3).

### 2.8. Bioinformatic Analysis

MiRNET software 2.0 analysis was employed to predict the target genes of the significantly upregulated miRNAs. Recovery of the results was obtained using the miRNET database (https://www.mirnet.ca/faces/home.xhtml (accessed on 12 May 2025)), which permits the integration of 11 existing miRNA–target prediction programs (TarBase, miRTarBase, miRecords, miRanda, miR2Disease, HMDD, PhenomiR, SM2miR, PharmacomiR, EpimiR, and starBase), while the Enrichr database was used to investigate the signaling in which miRNAs and their targets are involved.

### 2.9. Statistical Analysis

Statistical analyses were performed using the StataNow 18.5 software (StataCorp. 2023. Stata Statistical Software: Release 18.5. StataCorp LLC., College Station, TX, USA). Following the verification of data distribution and homogeneity via the Shapiro–Wilk and Levene tests, group differences were assessed using a two-way linear model that included treatment, time, and their interaction, with robust standard errors obtained via bootstrap resampling (500 iterations). The significance of the model effects was tested using the Wald statistic. For post-hoc analysis, pairwise group comparisons were performed using linear combinations of the model coefficients, with Sidak correction applied for multiple testing. Adjusted *p*-values < 0.05 were considered statistically significant. The results are presented as the mean ± standard deviation.

## 3. Results

### 3.1. Mg Alloy Effects on the Cell Viability of RAW Cells

**Figure 1:**
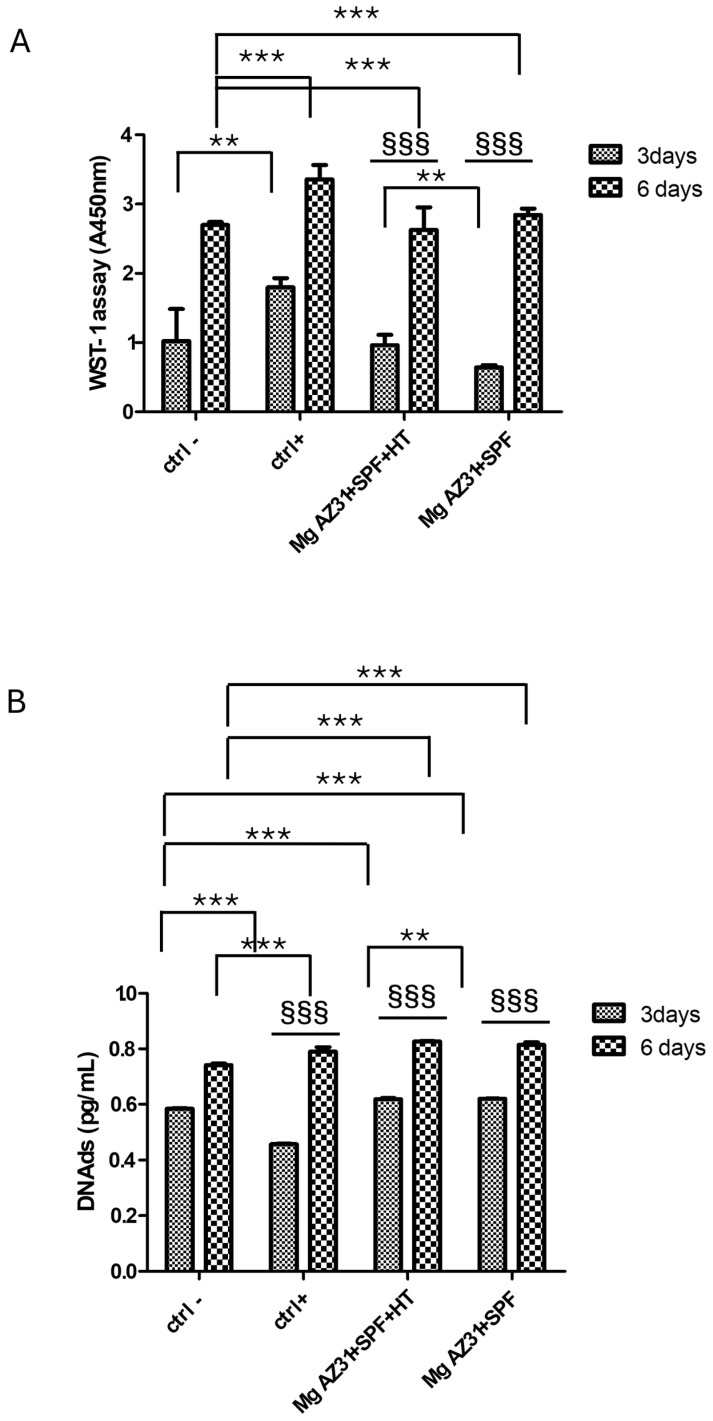
Viability evaluation on RAW 264.7 cells treated with Mg AZ31 extracts. Cell viability assays in hMSCs treated for 3 and 6 days with Mg AZ31+SPF and Mg AZ31+SPF+HT extracts: WST-1 (**A**) and PicoGreen (**B**). Data are expressed as a value of absorbance (**A**) at 450 nm or in quantity of DNAds (pg/mL). A two-way bootstrapped linear model test was used to evaluate the effect of the different Mg AZ31 extract treatments for the same extracts during the experimental period (§) or compared to other groups (*) (1 symbol, *p* < 0.05; 2 symbols, *p* < 0.005; 3 symbols, *p* < 0.0005). All experiments were triplicated, with the data expressed as the mean ± SD.

In order to investigate the biocompatibility of Mg AZ31+SPF and Mg AZ31+SPF+HT, an in vitro cell model of pre-osteoclasts was utilized. To this end, RAW 264.7 macrophages were treated with hrRANKL, Mg AZ31+SPF, and Mg AZ31+SPF+HT extracts, after which WST-1 and Pico Green assays were performed on days 3 and 6 of incubation. The data presented in Figure 1A demonstrate that the treatment of cells with hrRUNKL, Mg AZ31+SPF, and Mg AZ31+SPF+HT alloy extracts resulted in an enhancement of cell viability, with statistical significance differing between these treatments and the control group. Moreover, during the designated experimental timeframe, an increase in proliferation was observed in Mg AZ31+SPF (*p* < 0.0005) and Mg AZ31+SPF+HT (*p* < 0.0005). As demonstrated in Figure 1B, the quantification of DNA double-strand breaks (DNAds) corroborated the proliferation effects that had been demonstrated by metabolic activity. Notably, the Ctrl+ group exhibited a statistically significant increase during the experimental period (*p* < 0.0005). Based on this evidence, we investigated the effects of Mg extract treatments on the activation of osteoclast differentiation.

### 3.2. Effects of Mg Alloy Extracts on Pre-Osteoclast Marker Expression

**Figure 2:**
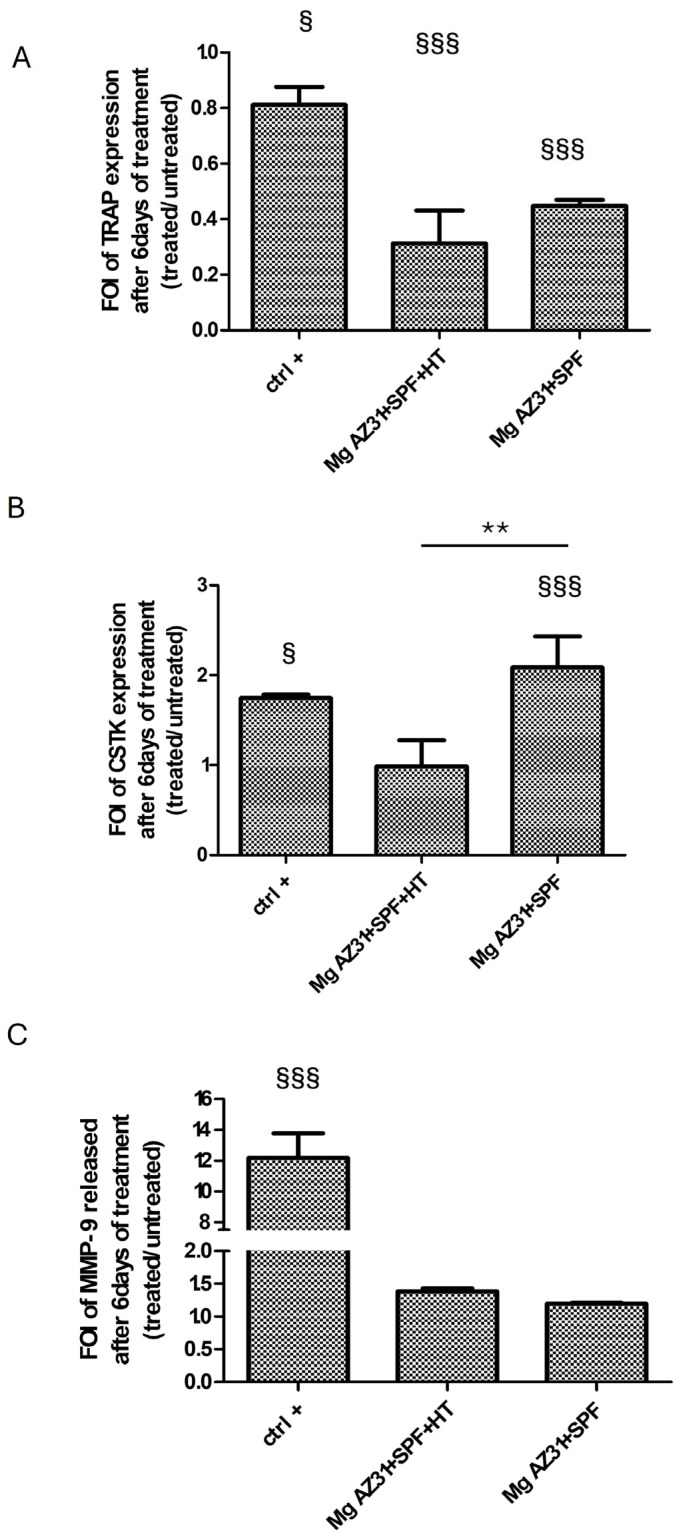
Effects of Mg AZ31 extracts on pre-osteoclast cells. qRT-PCR analysis of TRAP (**A**), CTSK (**B**) gene expression on RAW 264.7 cells treated for 6 days with Mg AZ31+SPF and Mg AZ31+SPF+HT extracts. Data are expressed as fold of change (FOI) in gene expression (2^−ΔΔCt^) that occurred in treatment vs Ctrl-. (**C**) MMP9 ELISA assay performed on the supernatant of RAW 264.7 cells treated with Mg alloys extracts or hrRANKL. A two-way bootstrapped linear model test was used to evaluate the effect of the different Mg AZ31 extract treatments for the same periods (*) or compared to Ctrl- (§) (1 symbol, *p* < 0.05; 2 symbols, *p* < 0.005; 3 symbols, *p* < 0.0005). All experiments were triplicated, with the data expressed as the mean ± SD.

To investigate the effect of Mg AZ31+SPF and Mg AZ31+SPF+HT extracts on pre-osteoclast activation, the expression of osteoclast genes, release of proteins, and, finally, cell morphology changes were evaluated. The mRNA expression of two key enzymes secreted by osteoclasts, during bone remodeling and mechanosensitive, such as TRAP (Acp5) and Cathepsin K (CTSK), was revealed through qRT-PCR analysis performed on RAW 264.7 cells after 6 days of maintenance in culture with hrRANKL or Mg alloy extracts. The data showed a decrease in TRAP expression for Mg AZ31+SPF+HT compared to the Ctrl- group (*p* < 0.0005), especially in cells treated with Mg AZ31+SPF extract (*p* < 0.0005). Meanwhile, an increase in CSTK mRNA expression was observed for RAW 264.7 cells cultured for 6 days in medium supplied with Mg AZ31+SPF extract (*p* < 0.0005) than Mg AZ31+SPF+HT (*p* = 0.004) or hrRANKL (*p* = 0.014) (Figure 2A,B). Regarding the evaluation of osteoclast signaling, we tested the release of metalloproteinase 9 (MMP9) protein after Mg extract or hrRANKL treatment. ELISA data revealed that RAW 264.7 cells showed a low level of MMP9 protein release compared to the Ctrl+ and Ctrl- groups after 6 days of culture with Mg AZ31+SPF (ns) and Mg AZ31+SPF+HT extracts (ns) (Figure 2C,D), while the Ctrl+ (*p* < 0.0005) displayed a significant release of MMP9 compared to Ctrl-.

**Figure 3:**
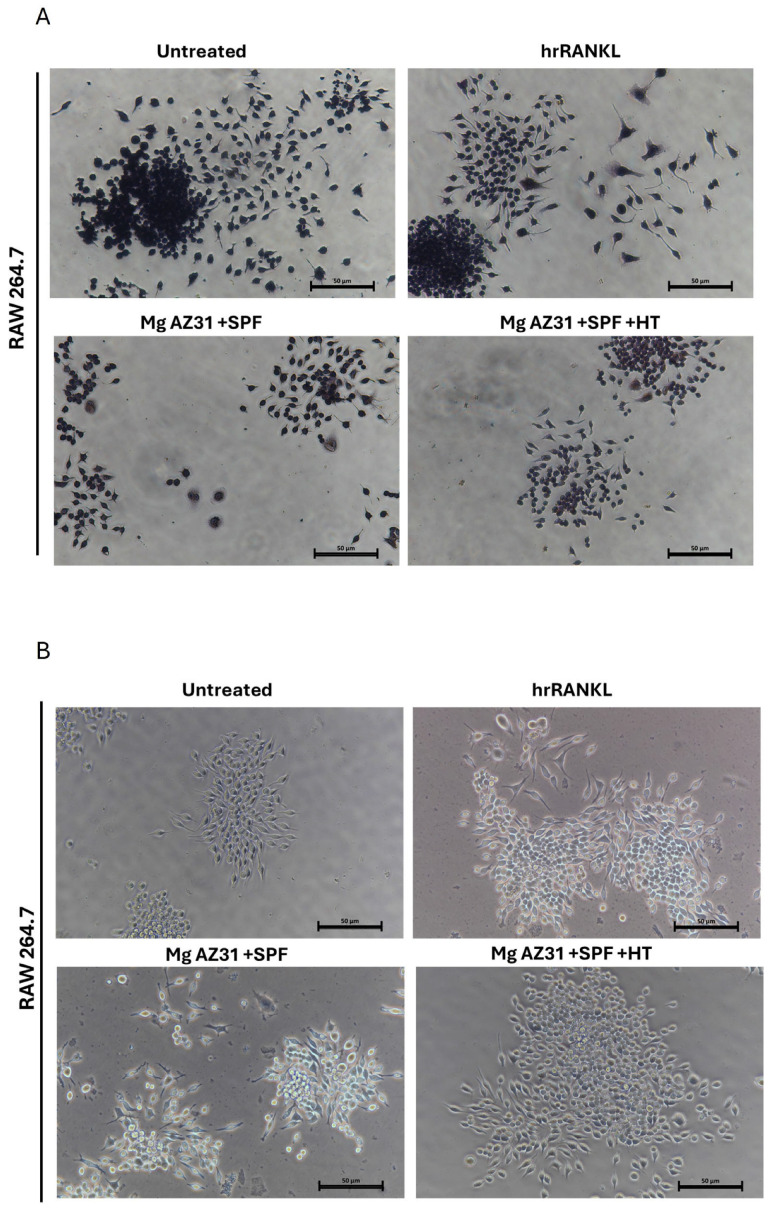
TRAP staining of osteoclasts on day 6. (**A**) TRAP staining assay of RAW 264.7 cells untreated or treated with hrRANKL (25 µg/mL), Mg AZ31+SPF extract, or Mg AZ31+SPF+HT extract for 6 consecutive days. Multinucleated TRAP+ cells containing more than three nuclei were considered mature osteoclasts. (**B**) Representative light microscope images using a Nikon Eclipse Ti microscope (10× magnification) of RAW 264.7 cells untreated or treated with hrRANKL (25 µg/mL), Mg AZ31+SPF extract, or Mg AZ31+SPF+HT extract for 6 consecutive days; scale, 50 μm.

The effects of Mg alloy extract on osteoclast differentiation were also evaluated through a TRAP staining assay. As reported in Figure 3, the treatments with Mg AZ31+SPF extracts induced an increase in the production of TRAP enzyme (A) and in terms of quantity of polynucleated cells (B) compared to Mg AZ31+SPF+HT alloy extracts. Obviously, a relevant increase in TRAP-positive cells and polynucleated cells was observed after RANKL treatments compared to the other groups.

### 3.3. Evaluation of Proteins Involved in Bone Remodeling Release After Mg AZ31+SPF and Mg AZ31+SPF+HT Extract Co-Cultures

**Figure 4:**
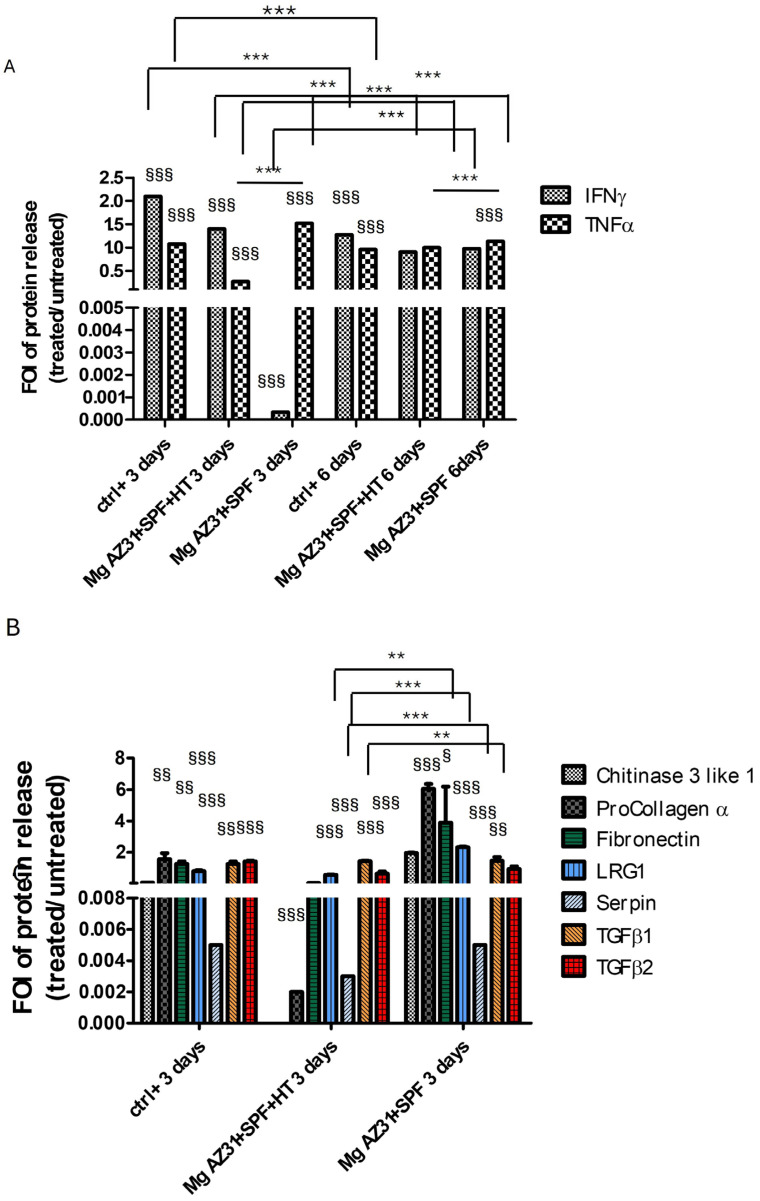

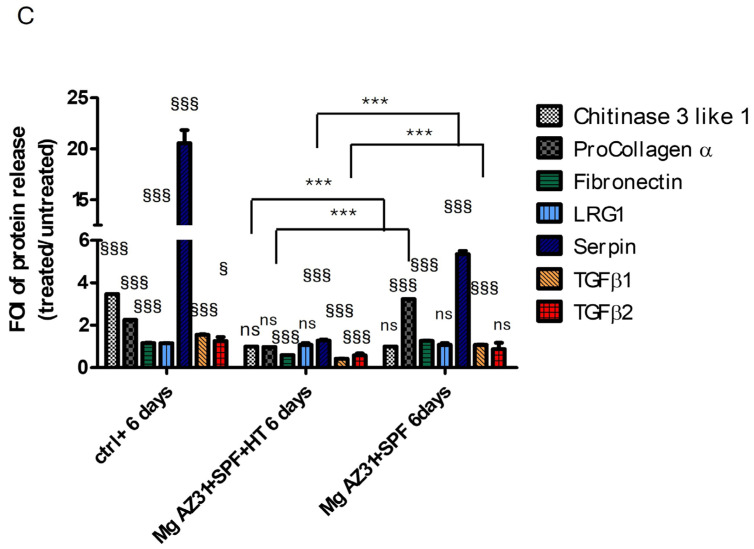
Analysis of the protein-released potential of Mg AZ31+SPF and Mg AZ31+SPF+HT extracts on RAW 264.7 cell supernatant soluble factors. BioPlex analysis of supernatant soluble factors (**A**) IFNγ and TNFα for 3 and 6 days of treatment and chitinase 3-like 1, procollagen α1, fibronectin, LRG1, serpin, TGF-β1, and TGF-β2 for 3 days (**B**) and 6 days (**C**) of treatment with Mg AZ31+SPF and Mg AZ31+SPF+HT extracts. Data are expressed as fold of induction (FOI) treated vs. Ctrl-. A two-way bootstrapped linear model was used to evaluate the effect of the different Mg alloys compared to Ctrl- (§) or the effects of treatment during the experimental period (*) or not statistically significant (ns) (1 symbol, *p* < 0.05; 2 symbols, *p* < 0.005; 3 symbols, *p* < 0.0005). All experiments were triplicated, with the data expressed as the mean ± SD.

Understanding the effects of hydrothermal treatment on Mg AZ31+SPF alloys in terms of the ability to induce the release of soluble factors is fundamental to knowing the maturation state of pre-osteoclast cells. Regarding this need, BioPlex assays were performed on supernatant recovery from RAW 264.7 cells after treatment with Mg AZ31+SPF and Mg AZ31+SPF+HT extracts or hrRANKL for 3 and 6 days to highlight the secretion of proteins involved in inflammatory signaling and bone healing (Figure 4). The data pertaining to IFNγ and TNFα (Figure 4A) revealed that IFNγ exhibited a relatively constant value of expression during the experimental period in the Mg AZ31+SPF+HT (day 3 vs. day 6, *p* < 0.0005) groups. However, a significant increase in expression was observed in the Mg AZ31+SPF (*p* < 0.0005) group after six days of treatment. The present study examined the release of TNFα. The data obtained from the Mg AZ31+SPF+HT group exhibited a slight yet statistically significant increase compared to the samples recovered after 3 days of treatment (day 3 vs. day 6, *p* < 0.0005). Furthermore, the analysis of the supernatant recovered from the Mg AZ31+SPF group revealed almost constant levels (*p* < 0.0005) during the treatment period.

Meanwhile, the analysis of chitinase 3-like 1 (ns), procollagen α1 (ns), fibronectin (*p* < 0.0005), LRG1 (*p* < 0.0005), serpin (*p* < 0.0005), TGF-β1, and TGF-β2 revealed a different secretion between the groups of cells maintained with or without Mg alloy extracts. After 3 days of maintenance in a culture with Mg AZ31+SPF+HT extract, RAW 264.7 cells displayed: (i) a lower expression of fibronectin, LRG1, TGF-β1, and TGF-β2 protein release than the Mg AZ31+SPF group; and (ii) the same release compared to the Ctrl- group (Figure 4B). Meanwhile, after 6 days of treatment with Mg AZ31+SPF+HT extract, the release of all tested soluble factors—chitinase 3-like 1 (ns), procollagen α1 (ns), fibronectin (*p* < 0.0005), LRG1 (ns), serpin (*p* < 0.0005), TGF-β1 (*p* < 0.0005), and TGF-β2 (*p* < 0.0005)—appeared lower than the Ctrl- group. Important increases in procollagen α1 (*p* < 0.0005) and serpin (*p* < 0.0005) were revealed after 6 days of treatment in the Mg AZ31+SPF group compared to Ctrl- (Figure 4C). Regarding the release of TGF-β1 and TGF-β2, a significant downregulation of TGF-β1 (vs. Ctrl- *p* < 0.0005; vs. day 3 *p* < 0.005) and TGF-β2 (vs. Ctrl- *p* < 0.0005) was observed in the Mg AZ31+SPF+HT and in Mg AZ31+SPF groups (for TGF-β1 vs. Ctrl-, *p* < 0.0005, and for TGF-β2 vs. Ctrl-, ns) during both experimental periods. Meanwhile, only TGF-β1 was modulated in a significant manner for Mg AZ31+SPF+HT (*p* < 0.0005) compared to Mg AZ31+SPF after 6 days of treatment, supporting our idea about the ability of hydrothermal treatments to mitigate the osteoclast differentiation of RAW 264.7 cells, which is consistent with the potential induction triggered by TGF-β2 that remained relatively constant during the experimental period.

### 3.4. Effects of Mg Alloy Extract Treatment on MiRNA Expression

**Figure 5:**
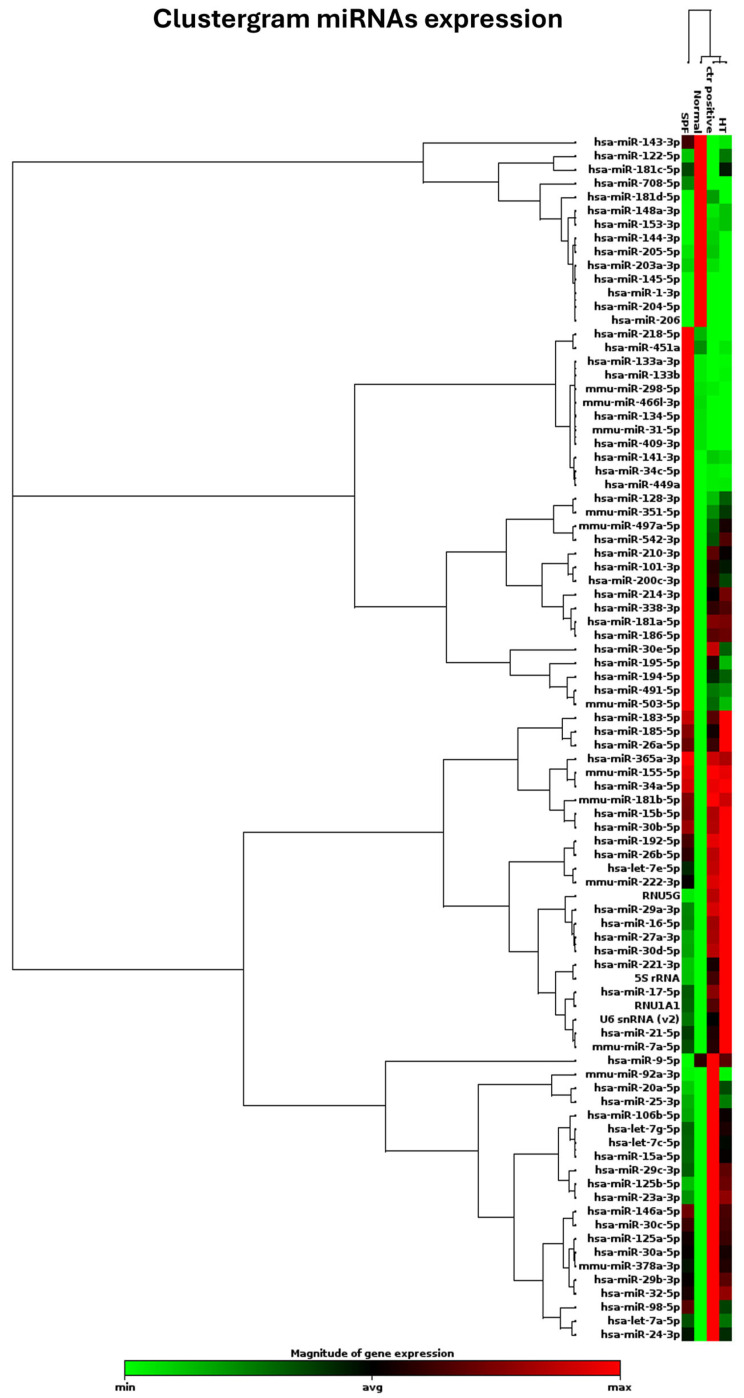
Clustergram of different miRNA expressions. Clustergrams were built using only those miRNAs showing significant changes. Clustergram analysis of miRNA expression revealed four distinct miRNA expression profiles. miRNA expression analysis was performed as described above, and clustergram analysis was based on the data described in Materials and Methods. The x-axis represents all groups listed, and the y-axis depicts miRNA expression profiles from all samples tested, normalized on the median value of 6 housekeeping genes. Each colored band represents the expression of a single miRNA from one group, with higher expression in red and lower expression in green.

It is understood that treatment can ensure cell vitality and probably mitigate activation of the osteoclastic differentiation process. However, the question remains as to whether hydrothermal treatment is also capable of modulating the miRNAs involved in bone regeneration without promoting the activation of osteoclasts but rather ensuring their maintenance in a state of physiological equilibrium. To accomplish this objective, an investigation was conducted on a panel of 82 microRNAs that were identified as playing a role in various processes that facilitate proper regeneration and regulate the inflammatory process. These phenomena are known to be altered under conditions of failed implant osseointegration or pathology. The present study investigated the differential expression of 82 microRNAs (miRNAs) triggered by Mg AZ31+SPF+HT and Mg AZ31+SPF in in vitro RAW 264.7 pre-osteoclast cell models through the qRT-PCR approach. The clustergram presented in Figure 5 provides a comprehensive overview of the data obtained. Differential expression of microRNAs was observed between the groups. A difference was observed between the Ctrl- and Ctrl+ groups, while a list of modulated miRNAs between the Mg AZ31+SPF and Mg AZ31+SPF+HT groups was obtained.

**Figure 6:**
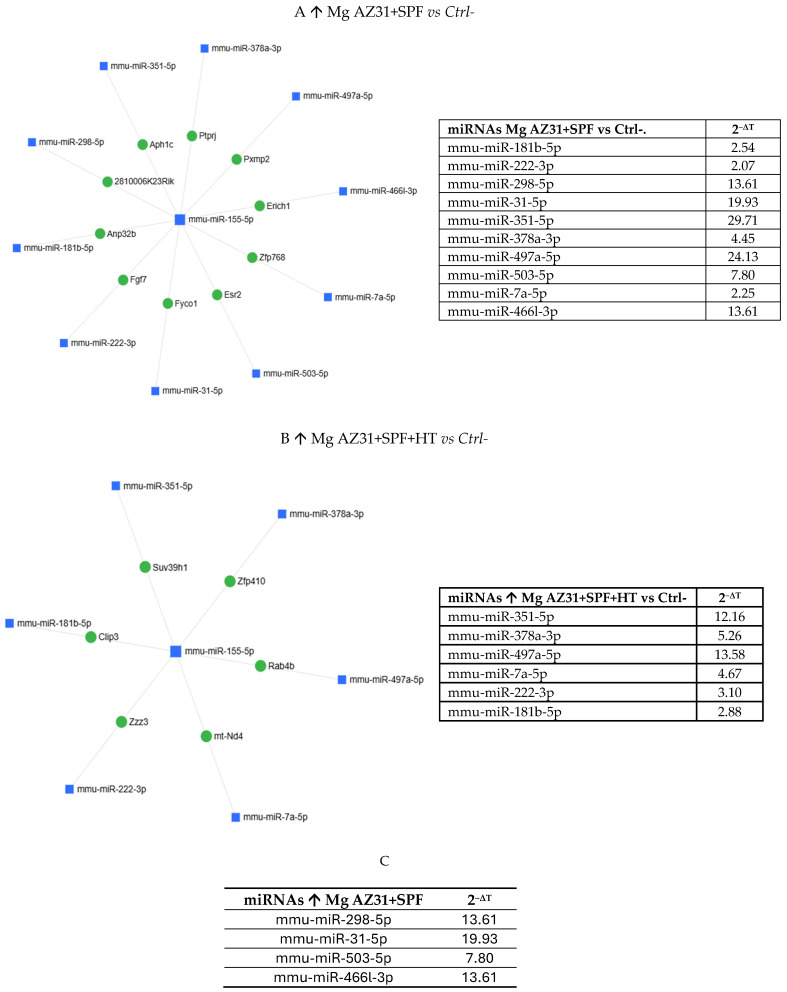
MiRNet analysis of miRNAs upregulated after treatment. (**A**) miRNAs upregulated (↑) in the Mg AZ31+SPF group or (**B**) Mg AZ31+SPF+HT group after 6 days of treatment. The left image shows the network of interaction between miRNAs and gene targets, while the right image shows the list of miRNAs upregulated and the value of expression obtained after normalization with the median value of 6 housekeeping genes. (**C**) List of miRNAs only increased (↑) in RAW 264.7 cells after Mg AZ31+SPF treatment that are not in common with other groups. The first column reports the name of the miRNA, and the second column shows the fold of induction (2^−ΔCt^) compared to Ctrl-.

Gene Globe software analysis underlined the miRNAs up- and downregulated in all experimental groups. Figure 6 reports the miRNAs upregulated in the Mg AZ31+SPF vs. Ctrl- groups (Figure 6A) and in the Mg AZ31+SPF+HT vs. Ctrl- groups (Figure 6B), while Figure 6C reports the six miRNAs not in common between the two groups.

**Figure 7:**
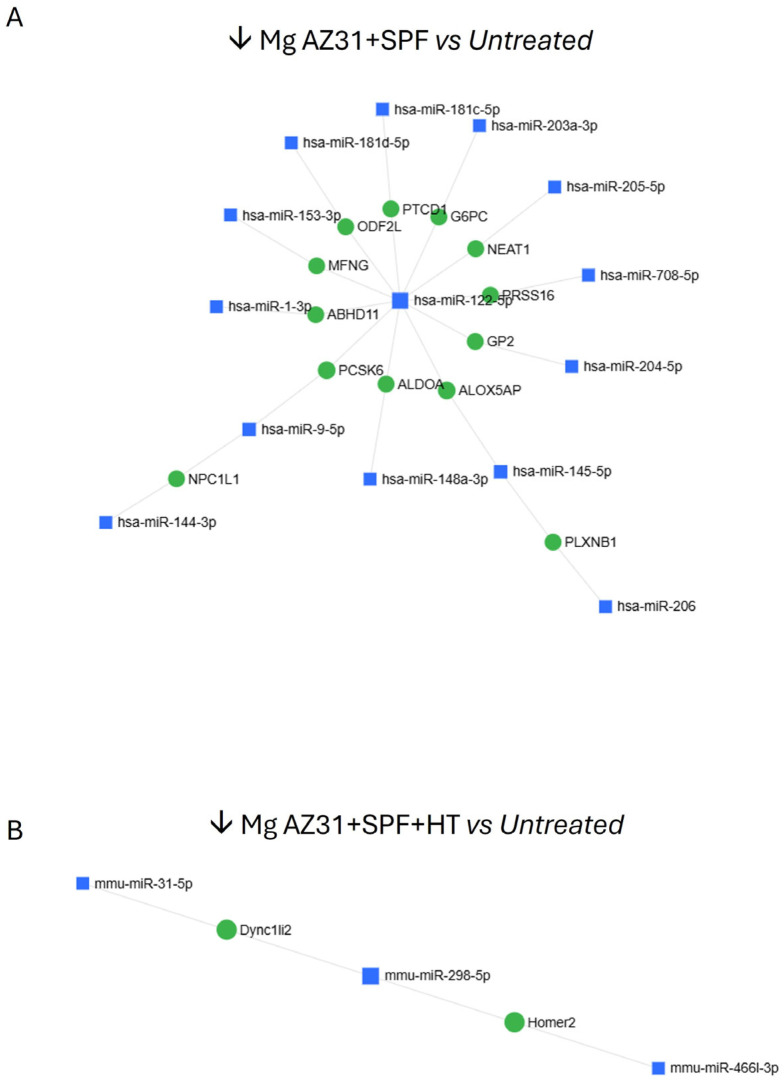
MiRNet analysis of miRNAs downregulated after treatment. (**A**) miRNA downregulated (↓) in the Mg AZ31+SPF group or (**B**) Mg AZ31+SPF+HT group after 6 days of treatment.

The downregulated miRNAs are illustrated in Figure 7 and detailed in Table 1, providing a comparison between Mg AZ31+SPF vs. Ctrl- (A) and Mg AZ31+SPF+HT vs. Ctrl-.

**Figure 8:**
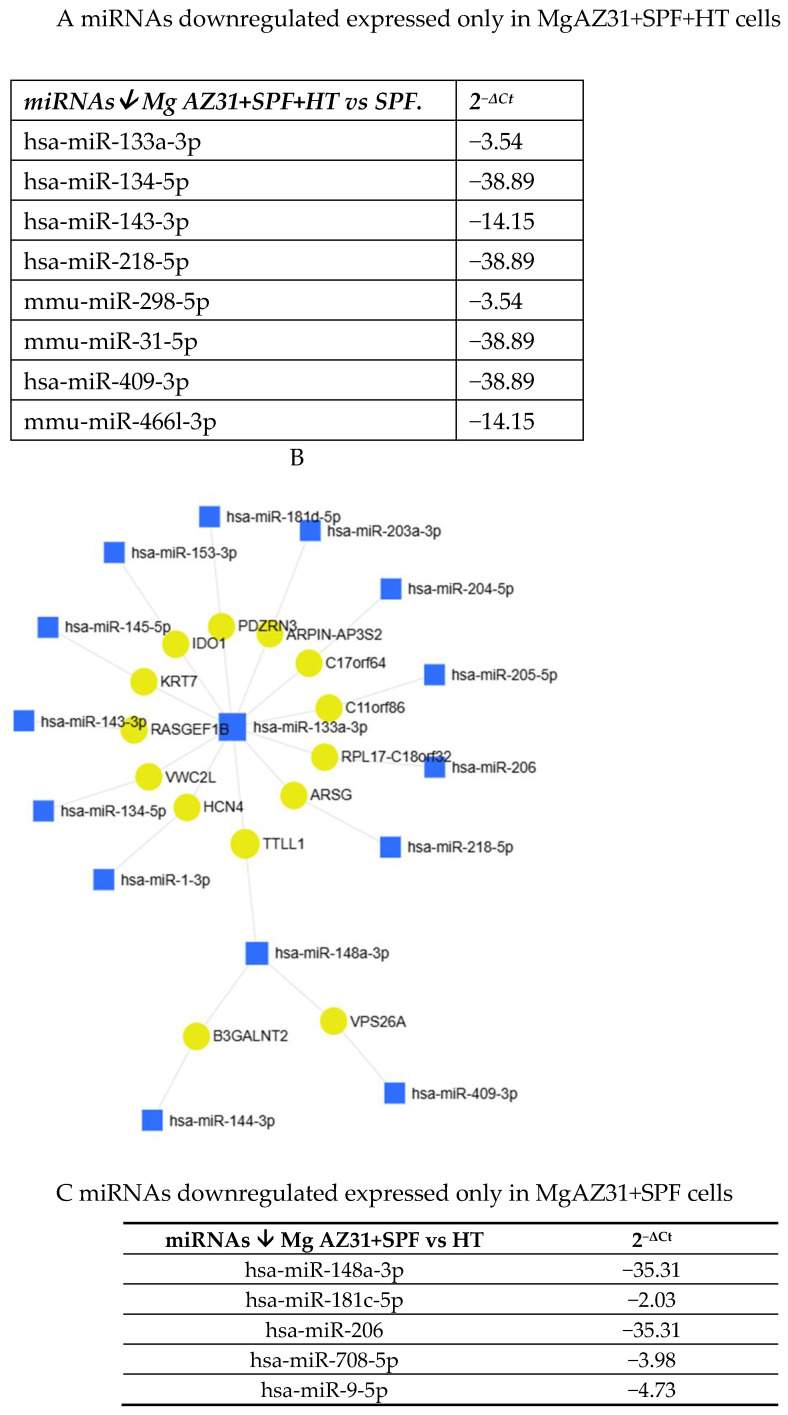
MiRNet analysis of miRNAs downregulated after treatment. (**A**) List of miRNAs downregulated (↓) only in RAW 264.7 cells after Mg AZ31+SPF+HT treatment. The first column reports the name of the miRNA, and the second column shows the fold of induction (2^−ΔCt^) compared to Ctrl-. (**B**) miRNet network of interaction between miRNAs and gene targets. (**C**) List of miRNAs downregulated (↓) only in RAW 264.7 cells after Mg AZ31+SPF treatment. The first column reports the name of the miRNA, and the second column shows the fold of induction (2^−ΔCt^) compared to Ctrl-.

Some of the identified miRNAs showed different expression in the samples. Specifically, they were either exclusively downregulated in Mg AZ31+SPF+HT-treated cells or exclusively downregulated in Mg AZ31+SPF-treated cells (Figure 8A–C).

Interestingly, network analysis of the miRNA data, followed by functional enrichment using a hypergeometric algorithm across multiple databases (e.g., KEGG, BP, and MF), revealed associations between miRNA expression and several diseases, including osteoarthritis, osteochondrodysplasias, osteosarcomas, musculoskeletal disorders, and rheumatoid arthritis. Using miRNet Analysis software, we investigated the possible targets of these identified miRNAs. This revealed that the miRNAs are involved in the regulation of genes that control immunity, inflammation, cell growth, cell differentiation, cell death, cell cycle, and metabolism. Subsequently, an investigation was directed toward the genes that impair osteoblast, osteoclast, and osteocyte function, leading to abnormal bone remodeling, and that are regulated by identified microRNAs. The use of network and functional enrichment analysis enabled the identification of the signaling pathways in which they are involved. Regarding the targets of the microRNAs that were found to be upregulated in the Mg AZ31+SPF group (see Supplementary Materials Figure S1A,B), it was determined that these were involved in the PI3–AKT pathways, FGF-2, and other intracellular signaling processes. Meanwhile, in the Mg AZ31+SPF-HT group, the targets of identified microRNAs were found to affect epigenetic regulation of gene transcription, chromatin organization, and the regulation of TNFR1 and TNF signaling (see Figure S1A–C). The preliminary overview suggested that, first, miRNAs induced by Mg AZ31+SPF treatment play a role in the pre-osteoclast morphology change; second, the miRNAs expressed by the Mg AZ31+SPF-HT group play a major role in the rearrangement of epigenetic gene regulation [22] to silence the expression of osteoclast markers in favor of osteoblast differentiation.

**Figure 9:**
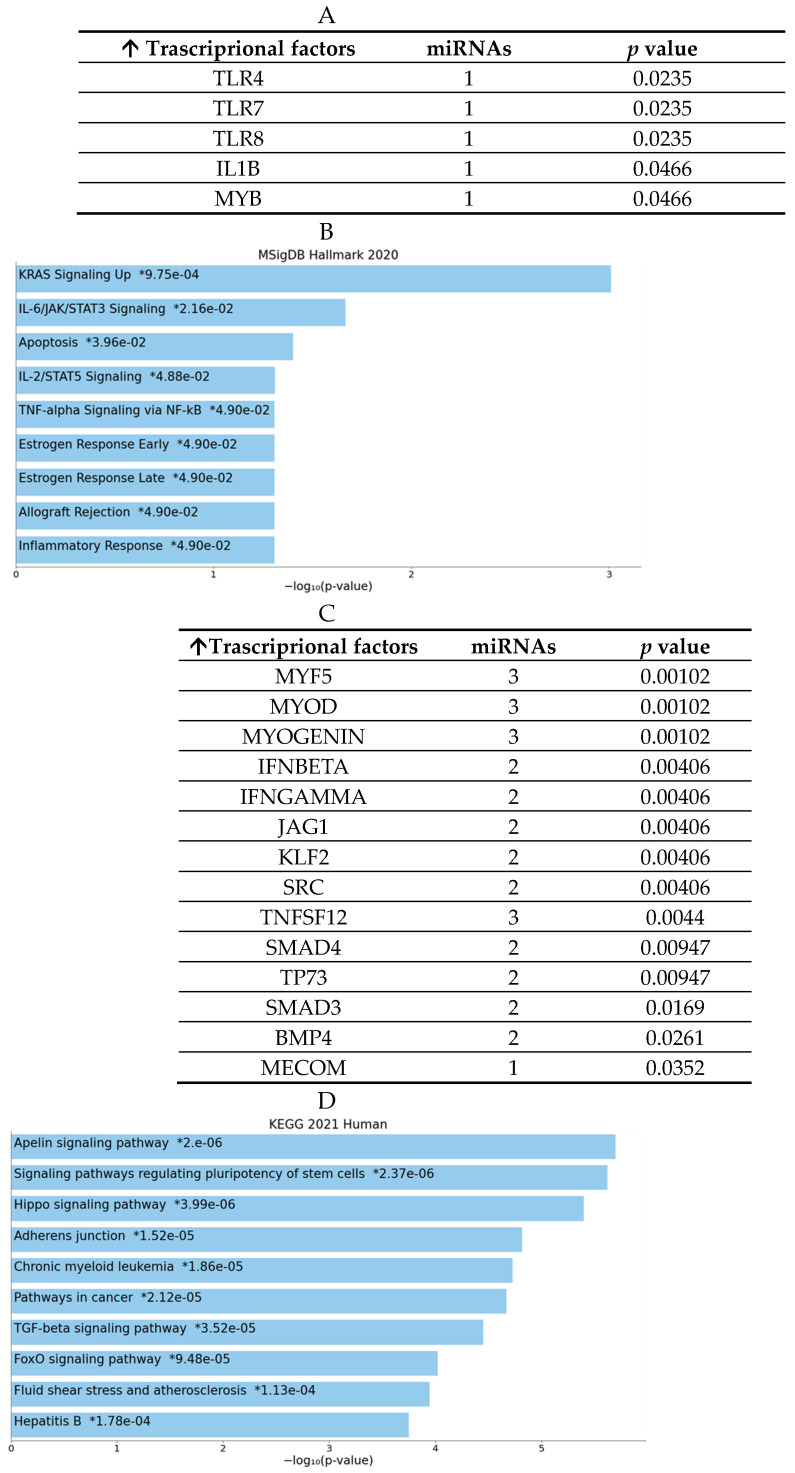
MiRNet analysis of downregulated miRNAs and target genes. (**A**) List of transcriptional factor (TF) targets of the downregulated miRNAs identified in the Mg AZ31+SPF group (name of miRNAs and their *p*-value). (**B**) Enrichment analysis using the MSingDB Hallmark database. The image reports the pathways in which the targets are involved. Bars are colored based on their *p*-value, while an asterisk next to a *p*-value indicates statistical significance. (**C**) List of TF targets of the downregulated miRNAs identified in the Mg AZ31+SPF+HT group (name of miRNAs and their *p*-value). (**D**) Enrichment analysis using the MSingDB Hallmark database. The image reports the pathways in which the targets are involved. Bars are colored based on their *p*-value, while an asterisk next to a *p*-value indicates statistical significance.

Regarding the list of potential targets of significantly downregulated microRNAs, several transcription factors (TFs) were investigated. Intriguingly, some of these had previously been examined using BioPlex assays (see Figure 9). In particular, the enrichment analysis revealed that the TFs modulated by the miRNAs identified in the Mg AZ31+SPF group are involved in IL-6 signaling, IL-2/STAT5 processes, FLT3 pathways, PI3K and AKT signaling, inflammation and estrogen responses, TNFα via NF-κB signaling, etc. (see Figure 9). Nevertheless, the targets of microRNAs that were downregulated in the Mg AZ31+SPF+HT group are implicated in Hippo signaling, adherence junctions, FoxO signaling, shear stress, the regulation of pluripotent stem cells, TGF-β signaling, adherent junctions, and so on (Figure 9C,D).

## 4. Discussion

Magnesium alloys have gained increasing attention in the orthopedic field due to their unique combination of biocompatibility, biodegradability, and mechanical properties closely resembling natural bone, making them highly promising materials for temporary implants and bone repair applications [23,24]. These alloys demonstrate a high degree of regenerative potential; however, their rapid in vivo degradation can result in pH changes, immune responses, and bone resorption, thereby compromising treatment efficacy. Current research is oriented toward optimizing material design and degradation control to mitigate these challenges, including the prevention of excessive local magnesium ion concentrations due to rapid degradation, the mitigation of gas accumulation around the implant, and the control of uneven degradation patterns. In relation to this observation, in vitro and in vivo studies have demonstrated that Mg deficiency inhibits the activity of osteoblasts and activates that of osteoclasts, resulting in an overall increase in bone resorption [25]. This is also true if we think that magnesium deficiency, due to diminished intake or impaired intestinal absorption, can affect the maintenance of normal bone and is correlated with the development or progression of diseases such as osteopenia and osteoarthritis [9,26]. Indeed, a pivotal consideration is the optimal concentration range of magnesium ions. A lower concentration of Mg ions has been demonstrated to enhance osteoblast (OB) activity, while an excessive concentration has been shown to completely inhibit osteoclast (OC) function. This results in impaired bone resorption and disrupted bone remodeling, thereby altering the balanced OC activity. In order to overcome this limit, it was recently demonstrated that the hydrothermal coatings of magnesium AZ31 alloys permit the reduction of this aspect in favor of the induction of osteoblast differentiation of hMSCs, the activation of osteoblast marker expression, and EMT signaling activation [27]. In consideration of the potential in vivo utilization of Mg AZ31+SPF+HT alloys, which is hypothesized to facilitate optimal fracture healing through the balanced activation of cellular responses that are physiologically stimulated at the implant site, the present study was conceived. The objective of this in vitro study was to investigate the response of osteoclast precursors in contact with HT–magnesium alloys.

Starting with the biocompatibility data, which highlighted an increase in metabolic ability and quantity of DNAds of the RAW 264.7 cells after treatment with both Mg AZ31+SPF and Mg AZ31+SPF+HT alloy extracts and no toxic effects during the experimental period, we investigated the effects of Mg extracts on the expression of osteoclast markers. The analysis of specific markers of osteoclast differentiation, such as mRNA expression of TRAP and CTSK and the quantification of MMP9 protein released, demonstrated an activation of osteoclast differentiation in the Mg AZ31+SPG group than Ctrl-, while slow modulations of these markers were shown by the Mg AZ31+SPF+HT group compared to Ctrl-. The results were confirmed by a TRAP staining assay, which revealed an increase in multinucleated osteoclasts observed in RAW 264.7 cells treated for 6 days with Mg AZ31+SPF extracts compared to the Mg AZ31+SPF+HT group.

To better understand the in vivo role of Mg alloys on the regulation of the microenvironment of the fracture around the site in which the alloys were implanted, we investigated the effects of treatment with Mg AZ31+SPF and Mg AZ31+SPF+HT extracts on the release of soluble factors involved in bone remodeling. In our previous study, we demonstrated that Mg AZ31+SPF+HT extract improves the release of cytokines involved in EMT signaling on hMSCs in an in vitro model. Herein, we tried to understand the possible link between some soluble factors released and the regulation of the balance between osteoblast and osteoclast activation. The analysis of the supernatant after 3 and 6 days of treatments demonstrated that after 3 days of treatment with Mg AZ31+SPF+HT extract, RAW cells enhanced the rate of TNFα (*p* < 0.0005) and IFNγ (*p* < 0.0005) released in comparison to the Mg AZ31+SPF group, while at the final time point, TNFα (*p* < 0.0005) showed a similar expression between the two groups and IFNγ showed no significant discrepancies. In addition, we analyzed the expression of different soluble factors involved in the regulation of the bone-resorbing of osteoclasts [28], such as TGF-β1, TGF-β2, procollagen type I alpha 1, chitinase 3-like 1, serpin, fibronectin, and leucine-rich repeats and glycosaminoglycan binding 1 (LRG1).

TGF-β signaling accelerates RANKL-induced OC differentiation [29,30], and recent evidence suggests that lower doses may promote differentiation, while higher doses inhibit OC differentiation. TGF-β1 and TGF-β2 have been demonstrated to play a “dual” role in the regulation of the activation of pre-osteoclasts, which are the precursor cells of osteoclasts [31,32,33]. Under certain conditions, TGF-β1 and TGF-β2 have been shown to promote the expression of receptors and transcription factors involved in osteoclast differentiation, thereby favoring the activation of pre-osteoclasts [31,32,33]. On the other hand, procollagen type I is not involved directly in OC differentiation but, as a structural component of bone, can interact with osteoclasts and potentially affect their activity [34]. Chitinase 3-like 1 (CHI3L1) is a glycoprotein involved in bone remodeling and inflammation, and it can promote or inhibit osteoclast differentiation depending on its interaction with other signaling pathways [35]. Meanwhile, serpin displayed a different role in OC activation: It acts as an inhibitor of proteases and plays a role in regulating the extracellular matrix, and it is necessary for osteoclast activity and bone remodeling. In addition, we investigated the expression of fibronectin and LRG1. Fibronectin is a structural protein of the extracellular matrix involved in cell adhesion and migration and in OC differentiation through interaction with cell surface receptors. LRG1 is a collagen receptor, involved in the inhibition of osteoclast formation and signaling through leukocyte-associated immunoglobulin-like receptor 1 (LAIR-1) ligand [34,36]. BioPlex analysis showed that the cells treated with Mg AZ31+SPF+HT extract released low levels of these described proteins during the experimental period compared to the Mg AZ31+SPF-treated cells. Nevertheless, all multiplex evidence underlined that the Mg AZ31+SPF+HT extract can orchestrate the release of soluble factors needed to favor bone integration in a shorter time than the Mg AZ31+SPF extract, suggesting a promotion of the osteo-microenvironment around the site of implant. However, it should be noted that, in vivo, the factors that influence these effects are different, including concentration, timing, interaction with other cytokines (e.g., RANKL and M-CSF), and the activation status of the target cells. Therefore, comprehension of the in vivo mechanisms activated by Mg HT alloys on the bone extracellular matrix is of central importance in terms of soluble factors released or signaling activated [32,37]. Nevertheless, a discrepancy between the findings of these simplified in vitro systems and the complex in vivo bone microenvironment persists.

These data prompted our interest to investigate the effects of Mg AZ31+SPF+HT extract on the microenvironment of implants. Recent data report the importance of miRNAs and lncRNAs in OC differentiation, bone regeneration and remodeling, tissue engineering, and biomarker identification. Overall, miRNAs play a significant role in bone remodeling by regulating the differentiation and function of both osteoblasts and osteoclasts. In particular, miRNAs are involved in several physiological and pathological processes controlling osteoclast differentiation and activity [14]. It is widely acknowledged that the expression of microRNAs is highly context- and stage-specific. At the six-day stage, osteoclasts reached their functional phenotype of bone resorption. Consequently, the microRNAs expressed at this stage reflected the mature osteoclast regulatory network. In line with this, in this study, we preliminarily identified the possible differences in miRNA expression on RAW 264.7 cells treated with Mg AZ31+SPF+HT and Mg AZ31+SPF extracts and hrRANKL for 6 days. qRT-PCR data revealed a different expression of 82 miRNAs between the groups of cells. Gene Globe analysis reported a strong difference in the expression of these miRNAs between Ctrl- and Ctrl+; while after treatment with Mg AZ31+SPF+HT and Mg AZ31+SPF extracts, the cells expressed several miRNAs in a different manner. Only 6 miRNAs were upregulated in the Mg AZ31+SPF+HT group and 10 in the Mg AZ31+SPF group compared to Ctrl-. Among these, for example, miR-31 is defined as one of the highly upregulated miRNAs in osteoclasts; it regulates cytoskeletal organization in osteoclasts by modulating RhoA expression, thereby facilitating optimal bone resorption activity [12]. Notably, in our study, miR-31 was downregulated in Mg AZ31+SPF+HT-treated RAW 264.7 cells compared to Mg AZ31+SPF-treated cells.

We found miR-133a-3p downregulation in Mg AZ31+SPF+HT-treated RAW 264.7 cells compared to Mg AZ31+SPF-treated cells. The circulating expression levels of miR-133a-3p were found to be upregulated in the plasma from osteoporosis versus normal post-menopausal women and correlated with bone mineral density (BMD) [38]. Additionally, it has been described that miR-148a-3p affects osteogenic differentiation by modulating the Wnt/β-catenin pathway via DKK1 regulation [39]. For instance, miR-148a-3p was overexpressed in osteoclasts after Mg AZ31+SPF+HT treatment. In the present study, we evidenced the presence of other microRNAs (miRNAs) in our analysis, which have also been described in the literature as being involved in bone metabolism processes. For example, miR-351 has been reported to affect osteogenesis by (+)-cholesten-3-one through targeting VDR [40], while the progression of osteoporosis also passes through the function of miR-206, which targets HDAC4 [41]. Furthermore, it has been demonstrated that miR-206 exerts a negative regulatory effect on the osteogenic differentiation of bone marrow mesenchymal stem cells by targeting glutaminase [42]. Finally, the synergistic effects of miR-708-5p and miR-708-3p contribute to the accelerated progression of osteoporosis [43].

The enrichment analysis revealed that the miRNAs upregulated in the Mg AZ31+SPF+HT group modified the expression of the genes involved in chromatin-modifying enzymes, chromatin organization, epigenetic regulation of gene expression, MET receptor recycling, negative regulation of rRNA expression by SIRT1, formation of WDR5-containing histone-modifying complexes, PKMT methylation of histone lysines, regulation of TNFR1 signaling, and TNF signaling. Meanwhile, the miRNAs upregulated in the Mg AZ31+SPF group are involved in the regulation of constitutive signaling by aberrant PI3K in cancer, PI3K AKT signaling in cancer, PI5P, PP2A, and IER3 regulation of PI3K AKT signaling, negative regulation of the PI3K AKT network, FGFR2b ligand binding and activation, PIP3 activation of AKT signaling, negative regulation of FLT3, intracellular signaling by second messengers, activated point mutants of FGFR2, phospholipase C-mediated cascade, and FGFR2 signaling. The Mg AZ31+SPF+HT group displayed a downregulation of 20 miRNAs/82 compared to Ctrl-, while the Mg AZ31+SPF group showed a decrease in expression of 14/82 miRNAs. However, it is more interesting that when comparing the extracts of alloys between them, it is possible to observe a difference in expression of only eight miRNAs/20 in the Mg AZ31+SPF+HT vs. Mg AZ31+SPF groups and five miRNAs/14 in the Mg AZ31+SPF vs. Mg AZ31+SPF+HT groups. The enrichment and miRNet analysis reported that the miRNAs downregulated after Mg alloy treatment are involved in the regulation of immunity, inflammation, proliferation, differentiation, apoptosis, cell cycle regulation, and metabolism signaling. In addition, the same analysis revealed that the targets of these two groups of miRNAs are transcriptional factors involved in, for the Mg AZ31+SPF+HT group, Hyppo signaling, adherence junctions, FoxO signaling, shear stress, epigenetic regulation, SIRT1 signaling, TNFα signaling, etc.; and for the Mg AZ31+SPF group, in FLT3 pathways, PI3K and AKT signaling, inflammation and estrogen responses, etc.

However, according to the findings of the gene enrichment analysis, the collective evidence suggests that MgAZ31+SPF+HT extract alloys stimulate bone formation not only by directly promoting osteogenesis of mesenchymal stem cells (hMSCs) [27] but also by mitigating the pre-osteoclast activation through the regulation of the release of soluble factors and MiRNA expression on the bone microenvironment, with potential beneficial effects on bone regeneration.

## 5. Conclusions

Although significant progress and numerous discoveries have been made in recent years regarding the use of magnesium (Mg) alloys for bone repair in medical applications, several fundamental scientific questions remain unresolved, particularly concerning the effects of Mg alloys on pre-osteoclast activation. This study aimed to provide a novel perspective on the effects of Mg alloys, with a specific focus on osteoclast activation following treatment. In summary, this in vitro study highlighted several key findings regarding the biological impact of hydrothermally treated Mg AZ31+SPF alloys:

1. Hydrothermal treatment does not compromise the viability of pre-osteoclast cells, thereby supporting its biocompatibility;
2. It modulates the differentiation of pre-osteoclast cells and does not increase the number of polynucleated cells post-treatment;
3. The treatment helps to maintain a balanced release of soluble factors involved in bone healing;
4. It regulates the expression of microRNAs (miRNAs) associated with bone regeneration and fracture repair, including those involved in osteoclast differentiation, inflammation, immunology, bone regeneration, and bone resorption through modulation of miRNA targets.

In pursuit of further scientific advancement and to deepen our understanding, we are planning additional studies to elucidate the cellular and molecular mechanisms underlying bone regeneration, with particular emphasis on the role of miRNAs identified through transcriptomic and secretome analyses. Ultimately, in vivo validation of the findings presented in this study will provide further insight into the molecules involved in bone regeneration and the performance of Mg AZ31+SPF+HT alloys as an implant material. Hence, future in vivo investigations will be conducted to substantiate and expand upon our current evidence.

## Figures and Tables

**Table 1:** List of miRNAs downregulated (↓) in RAW 264.7 cells after Mg AZ31+SPF (left) or Mg AZ31+SPF+HT (second right) treatment. The first column reports the name of miRNA and the second column shows the fold of induction (2^−ΔCt^) compared to Ctrl-.

miRNAs ↓ Mg AZ31+SPF+HT vs. Untreated	2^−ΔCt^	miRNAs ↓ Mg AZ31+SPF vs. Untreated	2^−ΔCt^
hsa-miR-122-5p	−3.54	hsa-miR-122-5p	−7.38
hsa-miR-133a-3p	−3.54	hsa-miR-144-3p	−35.31
hsa-miR-134-5p	−38.89	hsa-miR-145-5p	−35.31
hsa-miR-143-3p	−14.15	hsa-miR-148a-3p	−35.31
hsa-miR-144-3p	−38.89	hsa-miR-153-3p	−35.31
hsa-miR-145-5p	−38.89	hsa-miR-181c-5p	−2.03
hsa-miR-148a-3p	−7.07	hsa-miR-181d-5p	−35.31
hsa-miR-153-3p	−7.07	hsa-miR-1-3p	−35.31
hsa-miR-181d-5p	−38.89	hsa-miR-203a-3p	−6.56
hsa-miR-1-3p	−38.89	hsa-miR-204-5p	−35.31
hsa-miR-203a-3p	−14.15	hsa-miR-205-5p	−13.58
hsa-miR-204-5p	−38.89	hsa-miR-206	−35.31
hsa-miR-205-5p	−38.89	hsa-miR-708-5p	−3.98
hsa-miR-206	−38.89	hsa-miR-9-5p	−4.73
hsa-miR-218-5p	−38.89		
mmu-miR-298-5p	−3.54		
mmu-miR-31-5p	−38.89		
hsa-miR-409-3p	−38.89		
mmu-miR-466l-3p	−14.15		
hsa-miR-708-5p	−38.89		

## Data Availability

The datasets used and/or analyzed during the current study are available from the corresponding author upon reasonable request.

## Supplementary Materials

The following supporting information can be downloaded at: https://www.mdpi.com/article/10.3390/jfb16080303/s1, Figure S1. MiRNet analysis of up regulated miRNAs and target genes. (A) List of mRNAs targets of up regulated miRNAs identified on Mg AZ31+SPF group in the first column and on Mg AZ31+SPF+HT group for the second column. The targets are reported as follows: first name of mRNAs on the left of the list and the p value on the right. (B–C) Enrichment analysis using Reactome pathways database. The image reported the pathways in which the targets of miRNAs up regulated in Mg AZ31+SPF (B) group and in Mg AZ31+SPF+HT (C) are involved and its *p* value.

## Abbreviations

The following abbreviations are used in this manuscript:

HT	Hydrothermal treatment
Mg AZ31+SPF	AZ31 magnesium alloy manufactured via a superplastic forming process
Mg AZ31+SPF+HT	AZ31 magnesium alloy manufactured via a superplastic forming process and coated using a hydrothermal method
hMSCs	Human mesenchymal stem cells
M-CSF	Macrophage colony-stimulating factor
BMM	Bone marrow monocyte–macrophage
RANKL	Receptor activator of nuclear factor NF-KB ligand
lncRNAs	Long non-coding RNAs
miRNAs	MicroRNAs
3′-UTR	3′-untranslated regions
TNFa	Tumor necrosis factor alpha
IFNa	Interferon gamma
TGF-β1 and -β2	Transforming growth factor-β1 and -β2
DNAds	DNA double-strand
RT-qPCR	Real-Time Quantitative Reverse Transcription-Polymerase Chain
CTSK	Cathepsin K
MMP9	Matrix metalloproteinase 9
TFs	Transcription factors
LRG1	Glycosaminoglycan binding 1
CHI3L1	Chitinase 3-like 1
LAIR-1	Leukocyte-associated immunoglobulin-like receptor 1 ligand
MiRNET	miRNA-centric network
