# Supplementary material for: Hydrothermal Magnesium Alloy Extracts Modulate MicroRNA Expression in RAW264.7 Cells: Implications for Bone Remodeling
Source: J Funct Biomater. 2025 Aug 21;16(8):303. doi: 10.3390/jfb16080303 (PMC12387274; doi:10.3390/jfb16080303)
Supplement: Supplementary file 1 [file jfb-16-00303-s001.zip › jfb-3786884-supplementary.pdf]

Journal of
Functional Biomaterials

Supplementary files

Supplementary Figure S1

A

mRNAs targets of miRNAs ↑Mg AZ31+SPF	mRNAs targets of miRNAs ↑ Mg AZ31+SPF+HT
Aph1c	Clip3
Erich1	Rab4b
Esr2	Suv39h1
Fgf7	Zfp410
Fyco1	Zzz3
Ptprj	
Pxmp2	
Zfp768	

B ***Mg AZ31+SPF***

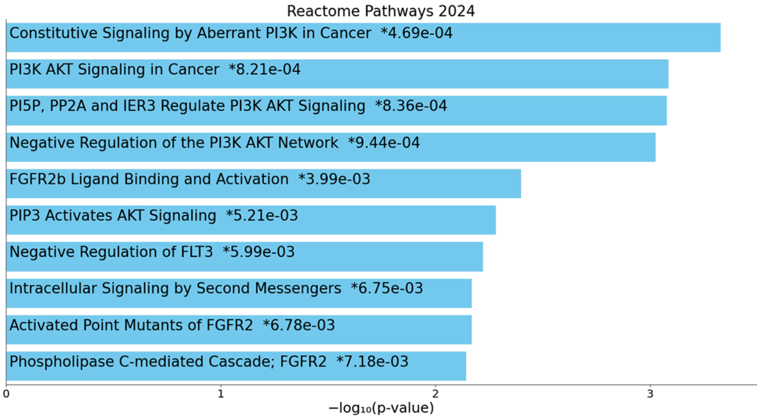

C ***Mg AZ31+SPF+HT***

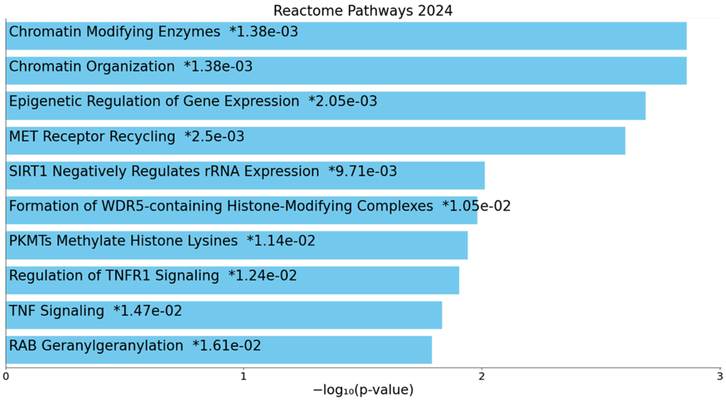

Figure S1. MiRNet analysis of up regulated miRNAs and target genes. (A) List of mRNAs targets of up regulated miRNAs identified on Mg AZ31+SPF group in the first column and on Mg AZ31+SPF+HT group for the second column. The targets are reported as follows: first name of mRNAs on the left of the list and the p value on the right. (B–C) Enrichment analysis using Reactome pathways database. The image reported the pathways in which the targets of miRNAs up regulated in Mg AZ31+SPF (B) group and in Mg AZ31+SPF+HT (C) are involved and its *p* value.
